# Characterising reward outcome signals in sensory cortex^☆^

**DOI:** 10.1016/j.neuroimage.2013.06.061

**Published:** 2013-12

**Authors:** Thomas H.B. FitzGerald, Karl J. Friston, Raymond J. Dolan

**Affiliations:** Wellcome Trust Centre for Neuroimaging, London WC1N 3BG, UK

**Keywords:** Credit assignment, Reward, Value learning, fMRI adaptation

## Abstract

Reward outcome signalling in the sensory cortex is held as important for linking stimuli to their consequences and for modulating perceptual learning in response to incentives. Evidence for reward outcome signalling has been found in sensory regions including the visual, auditory and somatosensory cortices across a range of different paradigms, but it is unknown whether the population of neurons signalling rewarding outcomes are the same as those processing predictive stimuli. We addressed this question using a multivariate analysis of high-resolution functional magnetic resonance imaging (fMRI), in a task where subjects were engaged in instrumental learning with visual predictive cues and auditory signalled reward feedback. We found evidence that outcome signals in sensory regions localise to the same areas involved in stimulus processing. These outcome signals are non-specific and we show that the neuronal populations involved in stimulus representation are not their exclusive target, in keeping with theoretical models of value learning. Thus, our results reveal one likely mechanism through which rewarding outcomes are linked to predictive sensory stimuli, a link that may be key for both reward and perceptual learning.

## Highlights

• Rewarding outcomes reactivate sensory regions involved in stimulus representation.
• Within these regions reward is not directed specifically to stimulus-coding neurons.
• This resembles a teaching signal predicted by reinforcement learning models.

## Introduction

Successful reward learning requires that an organism processes information about appetitive and aversive states, as well as assign causal responsibility for such states to antecedent events, that usually take the form of sensory stimuli. Whilst the first problem has been the subject of considerable neuroscientific study, the second, ‘credit assignment’ problem has been little explored in humans. One way in which the brain might perform a credit assignment is to direct a ‘teaching signal’, based on rewarding outcomes, to regions involved in stimulus processing (Friston et al., 1994; Roelfsema et al., 2010). Recently, several studies report evidence consistent with this, showing that rewarding feedback is associated with activity in sensory areas associated with stimulus processing, even in the absence of concurrent stimulation in that modality (Brosch et al., 2011; Pleger et al., 2008, 2009; Weil et al., 2010).

What is less clear is how populations of cells in sensory regions, targeted by an outcome signal, relate to those involved in stimulus representation. Supervised learning schemes, such as error back-propagation (Rumelhart et al., 1986), require generation of error signals tailored for each unit (Roelfsema and van Ooyen, 2005). This predicts a specific reactivation by reward feedback of units involved in stimulus representation. Such schemes are efficient but are considered to lack biological plausibility (Crick, 1989). Value learning models, by contrast, use a non-specific error signal that only modifies eligible connections; namely, those mediating the valuable outcome (Friston et al., 1994; Sutton and Barto, 1998) (visual stimuli within the last five seconds, for example). In a neurophysiological context, this predicts a non-specific input to sensory neurons to enable an associative increase in the strength of synaptic connections between recently active cells (Bailey et al., 2000; Calabresi et al., 2007; Izhikevich, 2007; Roelfsema et al., 2010). Critically, these two possibilities make different predictions about the relationship between spatial patterns of activity reflecting stimulus and outcome processing in stimulus-processing regions of the sensory cortex.

**Fig. 1:**
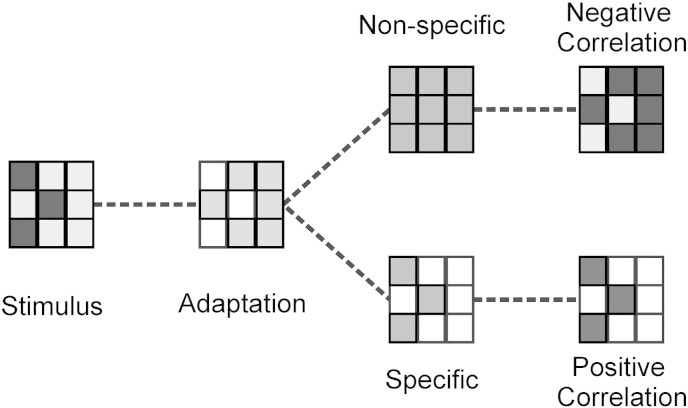
Cartoon illustrating the effects of non-specific and generalised reward feedback on the spatial correlations between stimulus and outcome activity. (Darker colours indicate greater activity/responsiveness) Stimulus processing produces an adaptation effect, manifest in a decreased responsiveness which is greatest in voxels with the strongest response to the stimulus. If reward feedback signals are non-specific, this leads to a negative spatial correlation between stimulus and outcome activity. If reward feedback signals are specific to those neurons involved in stimulus representation, there will be a positive spatial correlation between stimulus and outcome activity.

We tested predictions from these frameworks using fMRI adaptation. In brief, when two stimuli that activate the same neurons are presented in close temporal contiguity, the second stimulus produces a reduced BOLD response compared with an equivalent stimulus that does not activate the same population (Grill-Spector et al., 2006; Sawamura et al., 2006). Although the precise electrophysiological correlates remain unclear (Grill-Spector et al., 2006), this methodology has been used to probe stimulus representations across a range of distinct domains (Fang et al., 2007; Sawamura et al., 2006; Winston et al., 2004). We were interested in comparing neuronal responses to stimuli and their reward outcomes. To do this, we adopted a relatively new approach, based on spatial correlations within a region (Kriegeskorte and Bandettini, 2007; Kriegeskorte et al., 2008). We reasoned that if reward signals selectively reactivate sensory neurons involved in representing a preceding stimulus, then activations induced by the stimulus and reward should co-localise and their patterns, over voxels, should be positively correlated. Conversely, if reward outcomes activate sensory neurons in a non-specific fashion, recently-activated stimulus-specific populations should show adaptation and be less responsive to reward signals, resulting in the activation patterns due to stimulus and reward being negatively correlated (Fig. 1). Crucially, this negative correlation should occur in the context of an overall positive response to reward, distinguishing them from simple reward-induced deactivations.

**Fig. 2:**
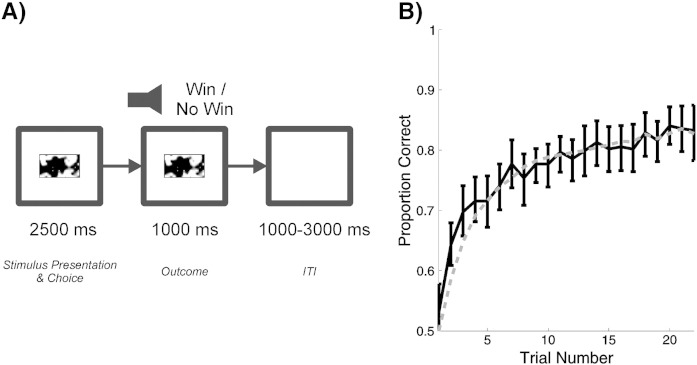
A: Reward learning task. Subjects were presented with a visual stimulus, and given 2500 ms to make one of two responses, which were rewarded according to fixed probabilities for each stimulus-action pairing. Outcomes were then signalled with two different sounds, which were presented for 1000 ms — followed by a jittered inter-trial interval (1000–3000 ms, uniform distribution). B: Learning curve, averaged across all cues and subjects. Subjects increasingly chose the objectively higher-valued option, indicating that they were able to acquire appropriate responses to the reward contingencies. (Solid line: learning curve based on observed choice behaviour. Dashed line: learning curve based on Q-learning models fitted to individual subject behaviour. Error bars indicate bootstrapped 95% confidence intervals for the observed choice behaviour).

To test our hypotheses, we analysed high resolution fMRI data (FitzGerald et al., 2012) collected whilst subjects performed an instrumental learning task with visual cues and auditory feedback (Fig. 2). Specifically, we examined spatial correlations within an area of visual cortex responsive to cues and rewarding outcomes.

## Materials & methods

### Subjects

Twenty six (ten female) right-handed subjects, age range = 19–28 years, all free of psychiatric or neurological disease, participated in the study. The study was approved by the Joint National Hospital for Neurology and Neurosurgery (University College London Hospitals NHS trust) and Institute of Neurology (University College London) Ethics Committee. The subjects were paid according to their performance during the task (from £21.80–£28.80).

### Stimuli & task

The subjects performed an instrumental learning task with visual cues and auditory feedback (FitzGerald et al., 2012) (Fig. 2). On each trial of the experiment, the subjects were presented with a visual cue consisting of a box with a coloured pattern, and made either a ‘left’ or a ‘right’ response by pressing a button on the corresponding keypad. After 2.5 s, they were played either a higher pitched ‘win’ sound, or a lower pitched ‘no win’ sound, each lasting for 1 s. The visual cue disappeared at the end of the sound. There was a variable inter-trial interval of 1–3 s between the trials. The subjects received 10 pence for each win.

Each cue had one of eight contingency types (win probabilities of [0.05 0.30], [0.05 0.55], [0.3 0.55], [0.4 0.9], with either *P*(Win|Chose Right) > *P*(Win|Chose Left) or the converse). Over the course of the experiment, each contingency type was repeated three times, using a total of 24 cues. The experiment was separated into blocks of 44 trials. In each block, two cues appeared in pseudo randomised order (we applied the constraint that no cue could be presented on more than three consecutive trials). Cues with identical or mirror image contingencies were never presented together in the same block. The subjects performed 6 blocks in each of two scanning sessions (12 in total). Each cue was presented in only one block. The subjects responded using two fMRI-compatible button boxes, one held in each hand.

### Behavioural analysis

Behavioural analysis was performed, as previously described, by fitting a *Q*-learning algorithm, incorporating a softmax decision rule (FitzGerald et al., 2012). *Q*-learning updates the values of individual stimulus action pairs *Q(s,a)* according to a reward prediction error weighted by a learning rate *α* (Watkins and Dayan, 1992).

$$Q\left(s_{t+1},a_{t+1}\right)=Q\left(s_{t},a_{t}\right)+\alpha \left(R_{t}-Q\left(s_{t},a_{t}\right)\right)$$

This algorithm generates trial-by-trial estimates of the values for each action (*Q_R_* and *Q_L_*), as well as the associated reward outcome prediction error (*RPE*). The softmax decision rule gives the probability of choosing an action *R* (*P_R_*) based on the difference in value between action *R* and action *L* (*Q_L_* − *Q_R_*), and the temperature parameter *τ* which determines the preference sensitivity between the two options.

$$P_{R}=\frac{1}{1+e^{\left(Q_{L}-Q_{R}\right)/\tau }}$$

The learning rate *α* and softmax temperature *τ* parameters were fitted individually for each subject using maximum likelihood (accuracy) estimators. Three subjects who reported using deterministic strategies on debriefing were excluded from the analysis, leaving a total of 23.

### fMRI data acquisition & preprocessing

Data acquisition and pre-processing were carried out as previously described (FitzGerald et al., 2012). Three-dimensional gradient-echo T2*-weighted echo-planar (EPI) images were acquired on a 3T Trio Siemens scanner with an isotropic resolution of 1.5 mm. 32 slices were acquired allowing data acquisition from a partial volume of thickness 48 mm that was angled and positioned in each subject to ensure coverage of the visual cortex, and reward-related regions such as the ventral striatum and ventromedial prefrontal cortex. In each session, 485 images were collected (~ 25 min each, two per subject). After discarding the first five images from the task sessions to allow for T1 equilibration effects, the fMRI time series were realigned and unwarped (Hutton et al., 2002) to correct for both static and motion-related distortions. For each subject, the T1-weighted structural and the mean whole brain EPI were then co-registered, and the partial volume EPIs co-registered to the whole brain EPI. Functional and structural data were then spatially normalised to MNI space using a DARTEL toolbox (Ashburner, 2007). For the purposes of ROI selection, the data were smoothed using a 4 mm^3^ FHWM Gaussian function. Respiration and heart rate were recorded using a breathing belt and pulse oximeter (Hutton et al., 2011).

### Region of interest selection

To identify functional ROIs in which reactivation might be expressed, we analysed (smoothed) data using a general linear model (GLM) containing events at (visual) cue onset times (*Cue*), and at (auditory) outcome time (*Outcome*). *Cue* was modulated by a parametric regressor encoding the absolute difference between the value of the two actions (|*Q_R_* − *Q_L_*|), and a binary regressor, reflecting the action (left or right) taken on each trial. *Outcome* was modulated by a parametric regressor encoding reward prediction error *RPE*. The six motion regressors produced by the realignment stage of preprocessing were included as regressors of no interest.

**Fig. 3:**
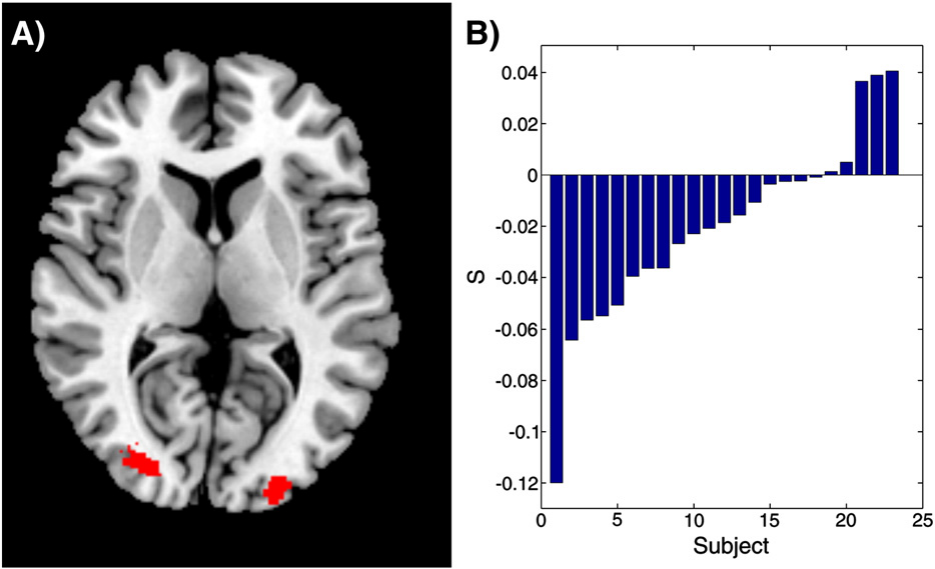
A: Functional ROIs for spatial correlation analysis. A conjunction analysis of group-level contrasts for the Cue and RPE regressors (thresholded at p < 0.05 uncorrected, with a minimum cluster size of 25 voxels) highlighted bilateral regions in the visual association areas (Brodmann area 18). These regions were used as functional ROIs for our spatial correlation analysis. B: Mean correlation between matched Cue/RPE images (S) for each subject. Overall, there was a significant negative correlation the two, consistent with the presence of a non-specific reward outcome signal in the region.

We selected regions for special correlation analysis by taking the overlap between voxels that showed significant positive responses to both *Cue* and *RPE* contrasts, thresholded at *p* < 0.05 uncorrected with a minimum cluster size of 25 voxels (84.38 mm^3^). We selected 25 voxels as a compromise between sensitivity and the need to exclude false positives, but the regions of interest, and hence our key results, were unaffected by varying the cluster threshold from 15 to 45 voxels. Additionally, very small clusters would be unlikely to yield reliable information about spatial correlations. This resulted in two clusters in bilateral visual association areas (Brodmann area 18) almost identical to those identified in a previous study (Weil et al., 2010) (Fig. 3) (Left cluster centroid [− 25 − 94 6], size = 253 voxels. Right cluster centroid [29 − 85 9], size 255 = voxels, MNI coordinates). Finally, a check analysis was carried out using a sphere of 6 mm radius placed outside the brain (centre [− 72 17 0]). No above-chance spatial correlations were observed in this region.

### Spatial correlation analysis

We first performed a general test of whether spatial correlations between patterns of activity at visual cue presentation and those at outcome presentation were positive, as predicted by stimulus-specific feedback signals, or negative as predicted by non-specific feedback signals. To do this, we analysed unsmoothed data using a GLM containing separate *Cue_i_*, *Difference in Value_i_*, *Action_i_*, *Outcome_i_* and *RPE_i_* regressors for each of the 24 cue types *i*, as well as the six motion regressors. We defined a parameter *S* as the mean of the correlation coefficients between *T*-statistics over voxels, in the regions of interest for the *Cue_i_* and *RPE_i_* contrasts (the effect of cue and RPE for each cue type). This allowed us to test for whether overall there were positive or negative spatial correlations between activity patterns to the cue and the rewarding outcomes. We used *T*-statistic images rather than contrast images for estimating spatial correlations as this down-weights the effect of noisy voxels, which has been shown to be advantageous in multivariate analysis (Misaki et al., 2010). *S* was then used as a summary statistic for group level inference performed using a two-tailed nonparametric Wilcoxon signed rank test. We first calculated a single value of *S* per subject, pooling data across both left and right ROIs, to maximise sensitivity. We then looked at the results for each side separately.

Importantly, our analysis is based upon the parametric *RPE* regressor rather than the regressor encoding the mere occurrence of the outcome event itself. This means that we have *de facto* regressed out any artefacts related simply to the proximity of the stimulus and feedback presentation events (for example through the use of a suboptimal haemodynamic response function). This means that our results are driven by the reward prediction error associated with each feedback event, which is orthogonal to the presence or absence of the event itself.

### Specificity of adaptation effects

To test whether the adaptation effects we observed were cue specific (driven responses in visual cortex that were specific to the cue being presented) or not (Kamitani and Tong, 2005). We first sought to establish that responses to cue presentation were cue specific. We did this by creating a GLM with separate regressors encoding cue onsets for each of the different cues, separated into the first and second half of the trials on which that cue was presented (so that for each cue, the model contained two regressors, one for the first half of the trials on which it appeared, and one for the second). We then calculated the spatial correlation between responses to the first cue presentation regressor and the second cue presentation regressor, and compared them to the mean spatial correlation with the regressors encoding the presentation of the other cues. Significance was assessed using a two-tailed Wilcoxon test as above.

Next we tested for evidence of cue-specificity in our adaptation effects. To do this, we compared the strength of the spatial correlations observed between matched *Cue* and *RPE* images (those corresponding to the same cue-type), and non-matched *Cue* and *RPE* images (those corresponding to different cue-types). Group level statistics were then calculated in the same way as for *S*. Finally, we tested whether the negative spatial correlations we observed were specific to paired stimuli and outcomes, by testing whether the mean correlation between non-matched *Cue* and *RPE* contrast images was significantly different from zero at the group level, using a two-tailed Wilcoxon signed rank test.

## Results

### Behaviour

As previously reported, both choice patterns and reaction times demonstrated that subjects used information about rewarding outcomes to modify their responses to visual stimuli (FitzGerald et al., 2012). In brief, subjects were more likely to select the ‘correct’ (higher valued) action at the end of a learning block than at the beginning, and showed a strong negative effect of absolute difference in value between the stimuli on reaction times, meaning that they responded more quickly when there was a greater disparity in the value of the actions available to them. The mean learning rate (*α*) across subjects was 0.284 (interquartile range: 0.191). The mean temperature parameter (*τ*) was 0.161 (interquartile range: 0.092). Averaged across subjects, the model predicted observed choices with 77.0% accuracy (*p* < 0.0001, Wilcoxon signed rank test).

### Spatial correlation analysis

Analysis of the spatial correlations between *Cue* and *RPE* provided our key measure of whether the outcome signals we observed in the sensory cortex show stimulus-specificity or not. A negative correlation provides evidence for an adaptation effect, suggesting a non-specific signal, whilst stimulus-specific signalling predicts the opposite. When we analysed spatial correlations across the combined visual cortices ROI, the group-level mean correlation between matched *Cue* and *RPE* images (*S*) was significantly less than zero (*μ* = − 0.02, *p* = 0.014, Wilcoxon signed rank test) (Fig. 3). This was also true for the right side considered individually (*μ* = − 0.024, *p* = 0.007), but the left side showed only a trend towards significance (*μ* = − 0.015, *p* = 0.101). We thus observed strong evidence of an fMRI adaptation effect, manifested in negative spatial correlations between stimulus and outcome activity (Fig. 1). This suggests, consistent with our hypothesis, that the reward outcome signalling we observed in visual areas involved in stimulus processing is non-specific, rather than being restricted to those neurons responding to the particular stimulus involved.

### Adaptation specificity analysis

We next sought to establish whether the observed adaptation effects occurred in neuronal populations which showed specific patterns of responding to visual cues, and, if so, whether these adaptation effects themselves were cue-specific. Spatial correlations between regressors encoding the same cue were significantly larger than those encoding different cues in the joint ROI (*μ* = 0.338, *p* < 0.001), and this was true for both the left and right ROIs considered separately (Left: *μ* = 0.337, *p* < 0.001. Right: *μ* = 0.340, *p* < 0.001). This indicates, in line with previous findings (Kamitani and Tong, 2005), that cue-evoked responses in this region of the visual cortex contain information about the specific visual stimulus presented.

Cue specific adaptation effects were assessed by comparing the difference between the size of the spatial correlation effects observed between *Cue* and *RPE* regressors corresponding to the same cue, and those corresponding to different (non-matched) cues. This difference was significantly negative across both ROIs (*μ* = − 0.021, *p* = 0.014), indicating that the adaptation effects we observed were indeed cue specific (driven by cue-specific visual responses). Again, the results were significant in the right ROI considered individually, (*μ* = − 0.022, *p* = 0.011), but the left side showed only a trend level effect (*μ* = − 0.018, *p* = 0.083). Overall, the spatial correlation between non-matched stimuli and outcomes showed no significant correlation, either calculated over both or when considered separately in the right (*μ* = 0.003, *p* = 0.693) or left (*μ* = − 0.003, *p* = 0.128) ROI.

Taken together, these results suggest that the adaptation effects we observed occurred in regions of the visual cortex which showed cue-specific patterns of responding, and that, in keeping with this, the adaptation effects we observed were driven by this cue-specific activity.

## Discussion

Linking sensory stimuli to outcomes they predict is critical for successful learning and adaptive behaviour. Previous studies have suggested that rewarding outcomes drive activity in sensory regions involved in stimulus representation (Brosch et al., 2011; Pleger et al., 2008, 2009; Weil et al., 2010), but have not addressed the specificity of this outcome targeting. Using spatial correlations, we found evidence suggesting that outcome signals are non-specific in their targeting of sensory regions.

By considering patterns of activity reflecting stimulus and outcome processing in regions of the sensory cortex that responded positively to both, we found strong evidence for a non-specific reward outcome signal in sensory regions that targeted both neuronal populations involved in stimulus representation and those which are not. In this sense our results are consistent with the predictions of value learning models, which posit the existence of a generalised feedback signal (Friston et al., 1994; Sutton and Barto, 1998), rather than one that specifically targets active units alone (Roelfsema and van Ooyen, 2005). Learning can still proceed efficiently in such a framework, despite the non-specificity of the error signal, provided that it exclusively affects the connection strengths between recently activated units (Bailey et al., 2000; Calabresi et al., 2007; Izhikevich, 2007). In other words, the credit assignment problem – induced by a non-specific RPE or value signal – is resolved simply by reinforcing recently activated stimulus-stimulus and stimulus-response links that precede valuable outcomes. Generally, models of this type of value dependent learning involve the modulation of associative plasticity in the form of a three way covariance rule (Friston et al., 1994).

At the same time, our results, together with those reported in other neuroimaging studies, suggest that information about rewarding outcomes can be selectively routed to, or amplified in, sensory regions associated with stimulus processing (Pleger et al., 2008, 2009; Weil et al., 2010). This suggests the existence of an additional mechanism for credit assignment, perhaps one mediated by attention, as recently proposed (Roelfsema and van Ooyen, 2005; Roelfsema et al., 2010). Indeed, the selective consolidation of eligible synapses by a non-specific reward prediction error signal may rest on exactly the same synaptic mechanisms that have been proposed recently to mediate top-down attentional effects; namely, a modulation of postsynaptic gain to presynaptic inputs (Feldman and Friston, 2010). In the context of predictive coding, this gain is thought to encode the precision or expected predictability of sensory information — for example, the offset of the visual cue that is preceded by the auditory outcome.

If outcome signalling were purely stimulus-specific, then we would expect to find a positive spatial correlation between stimulus and reward prediction error driven patterns of activity. The fact that we did not see this, and instead observed a strong negative correlation argues for the existence of non-specific reward outcome signalling in sensory areas. However, our results do not rule out the possibility that there is an additional stimulus-specific component to outcome signalling in the areas we tested, as might be predicted by supervised learning (Rumelhart et al., 1986) or augmented reinforcement learning (Roelfsema and van Ooyen, 2005; Roelfsema et al., 2010) theories. Such specificity might have been overshadowed by the strength of adaptation effects to non-specific outcome signalling at the short stimulus-outcome offsets used in our paradigm. Follow-up experiments with a longer gap between the stimulus and outcome could address this question. Additionally, it is possible that stimulus-specific outcome signalling might not have precisely the same effect on the BOLD signal as that of the coding of a visual stimulus (for example if it was expressed in changes in synaptic activity). In light of this, our results should be interpreted as evidence in favour of the existence of non-specific reward signals in sensory areas rather than evidence against the existence of stimulus-specific reward signals, since the two are by no means incompatible.

Two alternative interpretations of the negative spatial correlations we observed are that rewarding outcomes inhibit stimulus representations in the visual cortex, or that they preferentially activate cells not involved in stimulus representations. The former is extremely unlikely, given the overall positive effect of rewarding feedback on activity in the sensory cortex found in this and other studies (Brosch et al., 2011; Pleger et al., 2008, 2009; Weil et al., 2010). The latter is difficult to rule out, since it makes similar predictions to the generalised-feedback interpretation, but seems implausible under any theory of value learning of which we are aware.

Three (non-exclusive) models of repetition suppression have been put forward (Grill-Spector et al., 2006). In ‘fatigue’ models, neurons show a reduction in responding to the second stimulus that is proportional to the size of their response to the first one, perhaps through general firing rate suppression, or through reduced efficacy of particular synapses (Grill-Spector et al., 2006). In ‘sharpening’ models, neuronal populations become increasingly well-tuned, leading to a sharper representation of stimuli, and, under certain assumptions (Grill-Spector et al., 2006), an overall decrease in responding (Wiggs and Martin, 1998). ‘Facilitation’ models suggest that repeated stimuli produce smaller responses due to faster stimulus processing (James and Gauthier, 2006) or increased stimulus predictability (reduced prediction error) (Friston and Kiebel, 2009; Garrido et al., 2009; Rao and Ballard, 1999). Our results are most easily framed in terms of fatigue models, since it is difficult to see how either sharpening or facilitation would play a role here.

Our findings are of particular interest in light of the recent demonstration that outcome-correlated activity in the sensory cortex is dopaminergically modulated (Pleger et al., 2009). Given the anatomy of the dopaminergic system (Seamans, 2007), this is unlikely to be a direct effect, but instead is likely to be mediated by other regions more strongly innervated by the dopaminergic system, such as the ventral striatum or ventral medial prefrontal cortex. It is important to note that although in our analyses we model rewarding feedback in terms of reward prediction errors, the correlation between our *RPE* regressor and one simply reflecting rewards is strong in our study, so we make no strong claim about what precise parameter is signalled in the sensory cortex at outcome time.

Previous studies have focussed on outcome signals in the sensory cortex in the context of perceptual decision-making (Brosch et al., 2011; Pleger et al., 2008, 2009; Weil et al., 2010), whilst our study involved reward-based decision-making, in which the perceptual component is trivial. Since allowing reward information to boost neuronal plasticity is likely to be important both for perceptual (Roelfsema et al., 2010) and reward (Friston et al., 1994; Izhikevich, 2007) learning, it seems likely that similar or identical processes occur in both situations. Nonetheless, it is conceivable that they are different, and future studies could usefully test this.

Overall, our results suggest the existence of non-specific outcome signals in the sensory cortex that are consistent with the predictions of several models of value learning. Understanding how rewarding outcomes affect sensory processing is essential both for understanding the effect of reward on perceptual learning, and solving the problem of how the brain links sensory stimuli to the outcomes, rewarding or otherwise, that they generate. Our findings thus represent a step towards characterising the processes linking perception, learning and value.
